# Is the Clinician's Eye a Valid and Reproducible Tool for Diagnosing Patella Alta on a Lateral Knee Radiography?

**DOI:** 10.5435/JAAOSGlobal-D-20-00098

**Published:** 2020-07-02

**Authors:** Alex B. Vaisman, Andres N. Schmidt-Hebbel, Rodrigo K. Guiloff, Carlos Z. Valderrama, Sergio G. Arellano, Diego S. Edwards, Nicolas H. Rotman, Rafael R. Calvo, Nicolas V. Zilleruelo, David P. Figueroa

**Affiliations:** From the Department of Orthopedics and Traumatology, Clinica Alemana de Santiago (Dr. Vaisman, Dr. Schmidt-Hebbel, Dr. Guiloff, Dr. Valderrama, Dr. Arellano, Dr. Edwards, Dr. Rotman, Dr. Calvo, and Dr. Figueroa), Faculty of Medicine, Clinica Alemana de Santiago—Universidad del Desarrollo (Dr. Vaisman, Dr. Schmidt-Hebbel, Dr. Arellano, Dr. Edwards, Dr. Calvo, and Dr. Figueroa), and the Department of Radiology, Clinica Alemana de Santiago (Dr. Zilleruelo), Santiago, Chile.

## Abstract

**Introduction:**

Validity and reproducibility of the clinician's eye (CE) to diagnose patella alta (PA) on a lateral knee radiography (radiograph) is unknown.

**Methods:**

Cross-sectional study of 46 lateral knee x-rays. Three blind observers used CE, Insall-Salvati (IS), modified Insall-Salvati (mIS), and Caton-Deschamps (C-D) to determine patellar height. Sensitivity and specificity of each observer was compared with the musculoskeletal radiologist's C-D measurements. Intraobserver and interobserver agreement were assessed with intraclass correlation coefficient and Fleiss κ, respectively. Time needed to estimate patellar height for every method was recorded in seconds. Statistical differences between observers were calculated with a generalized estimating equation. Analysis of variance and post hoc Bonferroni test compared duration of each method (*P* < 0.05). Data were analyzed using Stata 15 (StataCorp).

**Results:**

CE, IS, mIS, and C-D's sensitivity and specificity values are as follows: 77%, 92%; 94%, 52%; 67%, 58%; and 53%, 89%, respectively. Intraclass correlation coefficient and Fleiss κ of CE, IS, mIS, and C-D values are as follows: 0.66 and 0.43, 0.88 and 0.68, 0.54 and 0.09, and 0.68 and 0.59, respectively. CE was the second most sensitive and most specific method for diagnosis of PA, with moderate intraobserver and interobserver agreement. IS was the most sensitive method with good intraobserver and interobserver agreement. CE was significantly faster (*P* < 0.05) than all other conventional radiographic ratios.

**Conclusion:**

CE's sensitivity increases with observer's experience and is highly specific. If normal patellar height is diagnosed, no other ratios are necessary, even in the less experienced clinician. Intraobserver and interobserver reproducibilities were moderate and only inferior to the IS ratio. In case patellar height is uncertain with the CE, the IS ratio is the most sensitive and reproducible method to confirm the diagnosis of PA.

Patella alta (PA) or high-riding patella refers to a condition where the position of the patella is high in relation to the femur, femoral trochlea, or tibia, determined on a lateral radiography (radiograph). It may be present in the acute setting after patellar tendon ruptures, as a normal condition in asymptomatic patients or result in anterior knee pain after recurrent patellar dislocations, Osgood-Schlatter disease, and chondromalacia patellae.^1,2^

More than 10 radiographic ratios have been described to estimate patellar height on a lateral knee radiograph. Direct measures are those ratios that estimate patellar height in relation to the femur, whereas indirect measures assess patellar height in relation to the tibia. The indirect more frequently used ratios are the Insall-Salvati (IS),^3^ modified Insall-Salvati (mIS),^4^ and Caton-Deschamps (C-D),^2,5,6^ but the literature is not clear to determine which method is considered the benchmark for diagnosis of PA. A wide range of definitions, measurements and cutoff values, anatomic variants, and nonstandardized radiographic protocols makes it difficult to define which ratio is more precise.^2^ This in addition to the fact that most of these measurements are time consuming. Knee surgeons and radiologists frequently use their clinician's eye (CE) as a subjective visual appreciation to rule out PA before objectively measuring the patellar height. This method has not been reported nor compared with other conventional radiographic ratios (CRRs), and therefore, its validity and reproducibility are unknown in the literature.

## Objective

The primary objective of this study was to report sensitivity, specificity, intraobserver, and interobserver agreement of the CE and three other CRRs as methods for radiographic diagnosis of PA. Secondary objectives are to compare the results between observers and the time of examination of the CE compared with the three CRRs.

## Hypothesis

CE's sensitivity, specificity, intraobserver, and interobserver agreement are comparable with IS, mIS, and C-D. More experienced surgeons are more accurate than less experienced surgeons to diagnose PA with all four methods. CE is less time consuming than the other three CRRs.

## Methods

### Study Design

**Figure 1:**
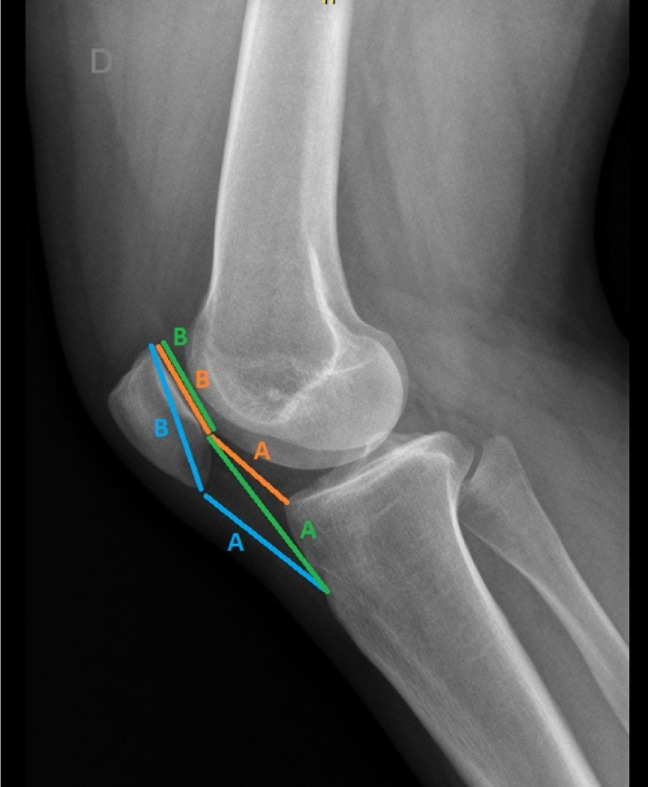
Radiograph demonstrating the conventional ratios on a lateral knee: IS (blue), mIS (green), and C-D (red). The IS ratio was calculated by dividing the length of the patellar tendon with the longest diagonal distance between the lower and the upper end of the patella. A value higher than 1.20 indicated PA. The mIS ratio was calculated by dividing the distance between the patellar tendon insertion and the lower end of the articular surface of the patella with the length of the articular surface of the patella. Values higher than 2.00 indicated a PA. The C-D ratio was calculated by dividing the distance between the peak of the visualized anterosuperior angle of the tibial plateau and the lower end of the articular surface of the patella with the length of the articular surface of the patella. Values higher than 1.30 indicated PA. C-D = Caton-Deschamps, CE = clinician's eye, IS = Insall-Salvati, mIS = modified Insall-Salvati, PA = patella alta.

**Figure 2:**
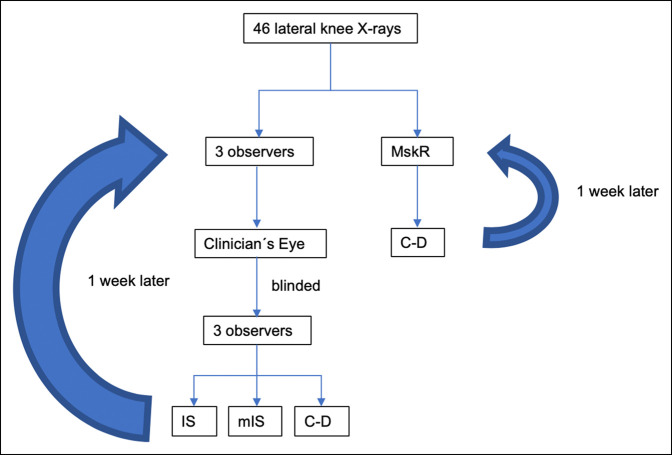
Flowchart demonstrating the radiographic analysis. C-D = Caton-Deschamps, IS = Insall-Salvati, mIS = modified Insall-Salvati.

A single center, cross-sectional study of 46 lateral knee radiographs from patients between 20 and 40 years of age were obtained from the institutional radiologic database (AGFA IMPAX 6, Mortsel, Belgium). Routine digital lateral radiography of the knee was performed with the patient lying on the affected side. The affected knee joint was flexed ideally to 30° with a support for the ankle and foot. The unaffected leg was positioned behind the affected leg. Inclusion criteria were complete superposition of both femoral condyles and assessment by the same trained musculoskeletal radiologist (mskR). Patients with degenerative joint disease, calcified patellar tendinitis, history of fractures or tendon ruptures, and previous knee surgeries were excluded from the study. The CE method was compared with the three most frequently reported CRRs: IS, mIS, and C-D.^2,4,7-9^ The CRRs ratios are detailed in Figure 1. Three observers (orthopaedic resident [OR], knee surgeon with less than 5-year experience [KS1], knee surgeon with more than 10-year experience [KS2]) independently evaluated all images and used the CE method, which consists of a subjective estimation of patellar height to confirm or rule out PA. Similar to the study where the mIS was validated,^4^ the validity of the CE was assessed comparing sensitivity and specificity of all three observers with the C-D measurements of a trained mskR, which was considered the benchmark. To assess intraobserver agreement, the mskR measured all images twice in a different order with a week of separation. Blinded to the previous CE evaluation, the three observers randomly reevaluated all images with IS, mIS, and C-D (Figure 1). The time needed to estimate patellar height for every method was recorded in seconds by each observer with a stopwatch. A week later, the same methodology was repeated in a different order to assess intraobserver and interobserver agreement (Figure 2).

### Statistical Analysis

All four measured ratios were assessed for the following two categorical values: presence or absence of PA. Sensitivity and specificity of CE, IS, mIS, and C-D were calculated in a contingency table and compared with the mskR's C-D measurements. Overall and individual intraobserver agreement (including the mskR) was calculated with the intraclass correlation coefficient (ICC). ICC values less than 0.5 are indicative of poor reliability, values between 0.5 and 0.75 indicate moderate reliability, values between 0.75 and 0.9 indicate good reliability, and values greater than 0.90 indicate excellent reliability.^10^ The interobserver reliability between the three orthopaedic observers were assessed with the Fleiss κ coefficient. The strength of agreement between observers was defined according to the guidelines of Landis and Koch^11^ as follows: poor, κ ≤ 0.20; fair, κ = 0.21 to 0.40; moderate, κ = 0.41 to 0.60; good, κ = 0.61 to 0.80; and excellent, κ = 0.81 to 1.00.

A priori power analysis was done. Bujang et al^12^ provided a guide to determine the minimum sample size required for estimating the desired effect size of ICC. According to this guide, the minimum sample size requirement for our study is 46 subjects when alpha is prespecified to be 0.05, power to be 0.90, and an ICC of 0.9 is expected between CE and C-D. Statistical differences between observers were calculated with a generalized estimating equation. Normality was assessed using the Shapiro-Wilk test. To compare the time needed to measure patellar height for every method, an analysis of variance and post hoc Bonferroni test were used. Statistical significance was set at 5% level (*P* < 0.05). Data were analyzed using Stata 15 (StataCorp). Institutional review board approval was obtained. Given the retrospective design and only use of anonymized radiographs, informed patients' consent was not deemed necessary.

## Results

### Validity and Reproducibility

CE, IS, mIS, and C-D's overall and average individual sensitivity, specificity, and intraobserver and interobserver agreement are resumed in Tables 1 and 2. The mskR's measured C-D had excellent intraobserver agreement (ICC of 0.93).

**Table 1:** CE, IS, mIS, and C-D: Average Individual and Overall Sensitivity and Specificity

Factor	CE		IS		mIS		C-D	
	Sensitivity	Specificity	Sensitivity	Specificity	Sensitivity	Specificity	Sensitivity	Specificity
Orthopaedic resident	65%	98%	92%	73%	46%	80%	48%	93%
Knee surgeon 1^a^	75%	89%	96%	48%	63%	68%	38%	86%
Knee surgeon 2^b^	81%	82%	96%	36%	94%	36%	75%	93%
Average	77%	92%	94%	52%	67%	58%	53%	89%

C-D = Caton-Deschamps, CE = clinician's eye, IS = Insall-Salvati, mIS = modified Insall-Salvati

a Less than 5-year experience.

b More than 10-year experience

**Table 2:** CE, IS, mIS, and C-D: Overall, Average Individual Intraobserver Agreement and Interobserver Agreement

Factor	Intraobserver Agreement		Interobserver Agreement
	ICC	95% CI	Fleiss κ
CE	0.66032	0.54269-0.77795	0.43
Orthopaedic resdent	0.617	0.49649-0.71331	
Knee surgeon 1^a^	0.826	0.7580-0.886290	
Knee surgeon 2^b^	0.524	0.41673-0.67805	
IS	0.88153	0.83692-0.92614	0.68
Orthopaedic resident	0.817	0.7380-0.86629	
Knee surgeon 1	0.942	0.91168-0.96178	
Knee surgeon 2	0.873	0.80852-0.91426	
mIS	0.54739	0.41673-0.67805	0.09
Orthopaedic resident	0.465	0.4122-0.5244	
Knee surgeon 1	0.477	0.4322-0.5623	
Knee surgeon 2	0.645	0.60448-0.6822	
C-D	0.67691	0.56417-0.78966	0.59
Orthopaedic resident	0.681	0.61716-0.76452	
Knee surgeon 1	0.663	0.54269-0.77795	
Knee surgeon 2	0.692	0.59335-0.79353	

C-D = Caton-Deschamps, CE, clinician's eye, CI = confidence interval, ICC = intraclass correlation coefficient, IS = Insall-Salvati, mIS = modified Insall-Salvati

a Less than 5-year experience.

b More than 10-year experience.

CE was the second most sensitive and the most specific method for diagnosis of PA, with moderate intraobserver and interobserver agreement. IS was the most sensitive method and also with the highest intraobserver (good) and interobserver agreement (good).

### Years of Expertise

#### Clinician's Eye

KS2 (with >10-years' experience) had a higher sensitivity with this method than the other two observers (Table 1). The OR and KS1 significantly underdiagnosed PA with this method in comparison to KS2 (*P* = 0.041 and *P* = 0.001) (Table 3). No statistically significant differences (*P* = 0.1) were found between the OR and KS1 (Table 4). KS1 had the highest intraobserver agreement (good). The OR's CE had the highest specificity.

**Table 3:** CE, IS, and C-D of the Orthopaedic Resident and Knee Surgeon 1 Using Knee Surgeon 2 as Reference

Factor	OR	95% CI	*P* Value
CE			
Orthopaedic resident	0.533	0.29-0.97	0.041
Knee surgeon 1	0.277	0.14-0.53	0.001
IS			
Orthopaedic resident	0.54	0.35-0.87	0.011
Knee surgeon 1	1.45	0.86-2.42	0.156
C-D			
Orthopaedic resident	1.22	0.71-2.11	0.46
Knee surgeon 1	2.33	1.37-3.94	0.002

C-D = Caton-Deschamps, CE = clinician's eye, CI = confidence interval, IS = Insall-Salvati, OR, odds ratio

**Table 4:** CE, IS, and C-D of the Orthopaedic Resident Using Knee Surgeon 1 as Reference

Factor	OR	95% CI	*P* Value
CE			
Orthopaedic resident	1.92	0.87-4.22	0.11
IS			
Orthopaedic resident	0.37	0.21-0.68	0.001
C-D			
Orthopaedic resident	0.53	0.29-0.94	0.029

C-D = Caton-Deschamps, CE = clinical eye, CI = confidence interval, IS = Insall-Salvati, OR = odds ratio

#### Insall-Salvati

No statistically significant differences (*P* = 0.156) were found between KS1 and KS2. The OR significantly underdiagnosed (*P* = 0.011 and *P* = 0.01) PA with this method compared with KS2 and KS1 (Tables 3 and 4).

#### Modified Insall-Salvati

Overall interobserver agreement was poor (κ = 0.09) with this method (Table 2). For this reason, no further comparisons were made between observers.

#### Caton-Deschamps

No statistically significant differences (*P* = 0.46) were found between the OR and KS2. KS1 significantly overdiagnosed PA in comparison to KS2 (*P* = 0.002) and the OR (*P* = 0.029) (Tables 3 and 4).

### Time of Measurements

CE was significantly faster than all other ratios (*P* = 0.0001) to estimate patellar height and diagnose PA (*P* < 0.05). No significant differences were found between IS, mIS, and C-D (Table 5).

**Table 5:** Time of Examination of all Methods Expressed in Seconds

Factor	CE	IS	mIS	C-D
Orthopaedic resident	17.3	143	149	161
Knee surgeon 1	20.4	164	190	185
Knee surgeon 2	25	197	175	176
Average	20.9	168	171.3	174

C-D = Caton-Deschamps, CE = clinician's eye, IS = Insall-Salvati, mIS = modified Insall-Salvati

CE was significantly faster (*P* = 0.0001). No differences between IS, mIS, and C-D (*P* = 0.99)

## Discussion

The main results of this observational study are that compared with IS, mIS, and C-D, the CE was the fastest, second most sensitive, and the most specific method to diagnose PA on a lateral knee radiograph. The clinical relevance of this study is that because of CE's high specificity, a PA diagnosed with this method does not require further measurements. CE is also a very sensitive method, particularly in trained observers. Therefore, it is a fast method to rule out PA in patients with normal patellar height. In daily practice most clinicians and radiologists do not do an objective patellar height measurement in every lateral knee radiograph, which would be very time consuming. Instead, a quick glance (CE) is performed intuitively, and eventually objectively measured when a normal patellar height is doubted. This study confirms that this intuition is correct, and the CE can be applied in the ambulatory healthy patients and could be helpful to decide whether an objective measurement is necessary for preoperative planification in patients with recurrent patellar instability. CE is also useful in the emergency department setting, where the attending surgeon or resident frequently has to make quick decisions and therefore can safely apply the CE method in the case of an acute trauma to the knee, before deciding to objectively measure the patellar height. If the clinician is uncertain of PA after applying the CE method according to the current study's results, the most sensitive and reproducible method to rule out PA is the IS method.

To our knowledge, this is the first study to assess validity and reproducibility of the CE compared with three CRRs. The reason why the authors chose the C-D as the benchmark over the IS method are the following: C-D is a simple, reproducible method validated in asymptomatic patients and reported sensitivity and specificity are similar to other CRRs.^9^ Experience is proven to increase reliability of patellar height measurements,^13^ and C-D is the mskR's preferred method to determine patellar height. In this study, C-D was measured by the same trained mskR and proved to have excellent intrarater reliability. Furthermore, the mIS method was validated using C-D as the benchmark.^4^ The authors state that the IS method has some major drawbacks; anatomically, it is the articular surface and not the patellar tip, which determines whether the patella is at a high-riding or normal position. Significant variations of the morphology of the patellae—for instance, a long distal patellar facet—may alter the IS ratio and would be falsely normalized.^4^ Previous surgeries to the tibia, specifically tibial tubercle osteotomies, may alter accurate measurements using the IS method.

The available research on the interobserver agreement of the most common patellar height ratios is scarce, with results varying from poor^8^ to excellent.^7,14^ CE had a moderate intraobserver agreement, higher than mIS, comparable with C-D but inferior to the IS ratio. CE's interobserver agreement was also moderate, higher than mIS but inferior to IS and C-D. A possible explanation for the moderate interobserver agreement is that one of our observers is a first-year orthopaedic resident, with far less experience than the other two observers. Verhulst et al^15^ reported a similar variability of observers but eliminated the less experienced observer from the statistical analysis. Smith et al^14^ assessed intraobserver reliability for different patellar height radiograph measurements and found the reliability of C-D to be better than Blackburn-Peel (BP) and IS. This differs to the current study, where the IS ratio had the best intraobserver reproducibility, followed by CE and C-D, whereas mIS had the lowest agreement. This study also confirms that all three measured CRRs reproducibility is variable and time consuming. Each method requires two measurements and a calculation within the normal ranges that are different among ratios and might be difficult to remember. Moreover, many authors agree that more than one method is needed to evaluate patellar height.^4,6,8,9,16^

Nizic et al^16^ applied a new method to diagnose PA on a lateral radiograph drawing a new proximal reference line. This line is drawn and moved upward to pass through the point where the posterior contours of the femoral diaphysis and the femoral condyles meet (the posterior reference point), parallel to the long axis of the egg-shaped superimposed femoral condyles. This method that was compared with IS, mIS, C-D, and BP was proved to be simple, faster, and more reproducibility than the aforementioned CRRs. No statistically notable differences were found between the binary interpretations of the measurements for the new reference line and the binary interpretations of the measurements for the most common patellar height ratios. This study differs with the current study because the authors did not report sensitivity or specificity of this new method, and although reproducible among trained observers, this was not assessed with less-trained observers that might have achieved a less favorable result.

As previously mentioned, this study compared results obtained between experienced, less experienced, and untrained observers. As expected, KS2 (with >10-years' experience) had a higher sensitivity with the CE method than both less experienced observers. However, the OR's specificity for CE was slightly higher than both knee surgeons. A possible explanation for this is that when an experienced clinician has a reasonable doubt with this method tends to over-diagnose PA and would use a CRR to objectively confirm the diagnosis. On the other hand, the untrained resident detects the more noticeable high-riding patellae but neglects the more difficult cases, improving specificity but compromising sensitivity. Given the high specificity of the CE among all observers, this method does not need further confirmation with other CRRs when normal patellar height is diagnosed, even with the less experienced observers. Experience is also an important factor when applying the IS ratio because the OR significantly underdiagnosed PA compared with the more experienced surgeons. The same was not confirmed with the C-D ratio, where significant differences were found between both more experienced surgeons. This could be due to the fact that KS1's preferred CRR is the IS ratio and seldomly applies C-D in his daily practice.

Recent studies^2,17^ describe good interobserver and intraobserver reproducibility for IS, BP, C-D, and patellar/trochlear index on MRI. Verhulst et al^15^ recently measured patellar height on radiographs, CT, and MRI in 48 patients who were treated for patellar instability. This study, similar to the current study's results, shows that the most reproducibility method to measure patellar height is the IS ratio measured on conventional radiographs and the patella-trochlear index on MRI.

**Figure 3:**
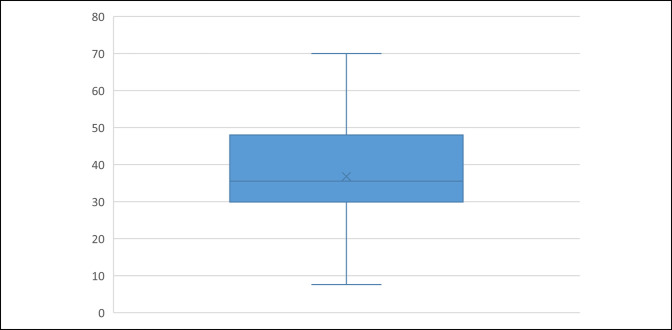
Box-and-whisker plot demonstrating the values of the angle of knee flexion spreading across the range between 7.6° and 70°.

A possible limitation of the current study is the variable angles of flexion of the analyzed x-rays, which could lead to imperfect measurements. Most studies report this as a limitation,^2,4,6,9,15,16^ but in current clinical practice, most routine lateral radiographs of the knee are performed within a range of knee flexion. For example, Seyahi et al^18^ reported that the lateral knee radiographs vary in their institution between 20° and 45°. However, Caton et al^19^ reported that the patellar height can safely be measured in a range of knee flexion between 10° and 80°, which is very similar to the range of flexion angles seen in the current study (Figure 3). Another limitation of the current study is that it was done in a white population in Chile, where cutoff values of patellar height have not yet been described. Althani et al^20^ demonstrated that the cutoff value for diagnosing PA in a Saudi population was 1.5 for the IS ratio and not 1.2 because it originally was described in a European population. Further studies are needed to confirm whether the cutoff values in Chilean patients differ from those of European and American populations.

## Conclusion

The CE's sensitivity increases with observer's experience and is highly specific. If normal patellar height is diagnosed, no other CRRs are necessary, even in the less experienced clinician. Intraobserver and interobserver reproducibilities were moderate and only inferior to IS ratio. In case patellar height is uncertain with the use of the CE, IS ratio is the most sensitive and reproducible method to confirm the diagnosis of PA.
